# Supplementary material for: Are carbon emissions trading and green financial instruments synergistic? -Comprehensive quantitative research based on content analysis
Source: PLoS One. 2024 Mar 7;19(3):e0298601. doi: 10.1371/journal.pone.0298601 (PMC10919601; doi:10.1371/journal.pone.0298601)
Supplement: S1 Data — (ZIP) [file pone.0298601.s001.zip › Data/地级市Z_F工作报告.zip!/╣π╬≈/┴°╓▌╩╨/└┤╫╘╣1⁄2╓┌║┼í╛┬φ┐╦╩2╛▌═°í┐_2015.docx]

各位代表：

现在，我代表市人民政府向大会作政府工作报告，请予审查，并请各位政协委员和列席会议的同志提出意见。

　　一、2014年工作回顾

　　过去的一年，面对复杂严峻的国际国内形势 、经济下行压力加大和超乎预料的困难，全市各族人民在自治区党委 、政府和市委的坚强领导下，深入学习贯彻党的十八大 、十八届三中 、四中全会精神，坚持稳中求进工作总基调，稳增长 、调结构 、促改革 、惠民生 、优环境，立足当前，着眼长远，凝心聚力，攻坚克难，努力推动各项事业取得新进展新成效，经济社会发展稳步前进，宜居宜业柳州蓬勃发展，人民生活更加美好。

　　——经济运行稳中有进。预计全市地区生产总值2120.8亿元，增长8.5%；工业总产值4406.0亿元，增长10.0%；财政收入316.6亿元，增长11.1%；固定资产投资1765.5亿元，增长16.0%。经济运行保持在合理区间，工业总产值 、财政收入 、固定资产投资继续保持两位数较快增长，经济质量和效益进一步提升，为今后发展奠定了扎实基础。

　　——人民生活持续改善。全市公共财政用于民生领域支出206.2亿元，占全市公共财政支出比重78.9%。全市城镇新增就业人数5.91万人，农村劳动力转移就业5.97万人，城镇登记失业率3.97%，居民消费价格涨幅控制在2.6%，就业 、物价形势基本稳定；城镇居民人均可支配收入26669元，增长9.5%；农民人均纯收入8660元，增长13%，人民生活水平进一步提高。

　　——结构调整成效明显。第三产业增加值643.6亿元，增长10.0%，超过二产1.6个百分点；社会消费品零售总额851.0亿元，增长12.2%，消费增长总体稳健；工业产业结构进一步优化，战略性新兴产业规模以上工业产值达357.0亿元，占全市比重8.3%，比上年提高1.2个百分点。

　　——社会事业加快发展。人口自然增长率7.35‰，控制在自治区下达的指标之内；学前教育三年毛入学率75.8%，比上年提高1.8个百分点；新农合 、城镇职工医保 、城镇居民医保参保率均达98.5%以上；为民办10件实事全面完成，社会保持和谐稳定。

　　一年来，我们突出抓了以下几个方面工作：

（一）着力推动转型升级，提升经济发展水平调结构促转型，加快新型工业化进程。深入实施“创新驱动，转型升级，工业再上新台阶”三年计划，产业结构 、产品结构进一步优化，汽车产业引领作用突出，产值突破2000亿元，产量超过200万辆，占全国比重达9%，成为全国第五个汽车年产量过200万辆的城市；上汽通用五菱 、东风柳汽产值分别迈上700亿元 、200亿元新台阶，宝骏730、景逸S50等新产品上市，柳州微车 、MPV销量继续领跑国内市场。冶金 、机械产业有效应对市场疲软，柳钢钢材产量达1300万吨，中高端钢材占总量50%以上；柳工大吨位装载机继续保持市场占有率第一。工业质量与效益进一步提升，全市规模以上工业总产值 、利税总额分别增长10.3%和14.0%，园区规模以上工业产值2400亿元 、占全市比重达56%；规模以上工业企业万元工业增加值能耗下降7.0%。创新能力不断增强，新增17家国家高新技术企业 、1家国家技术创新示范企业，大型企业生产装备自动化和半自动化率达90%。工业发展后劲持续增强，完成工业更新改造投资898亿元，增长15.7%，上汽通用五菱宝骏基地（二期） 、东风柳汽发动机等项目开工建设，玲珑轮胎（一期）试生产。

　　优布局促调整，加快现代服务业培育。万达 、华润 、恒大等服务业重大项目开工建设，完成投资40多亿元。梦之岛百货 、银泰城 、乐和城等大型商业综合体建成开业。现代物流发展初显成效，钢铁物流园销售收入突破100亿元，宁铁柳州汽车工业物流中心投入运营，柳州医药现代物流配送中心（一期）基本建成，新柳邕农产品批发市场（一期）主体完工。电子商务产业发展提速，桂中海迅柳北物流基地电子商务示范园区成为广西电子商务示范基地，已有25家电子商务企业入驻。旅游名城加快建设，百里柳江、三江程阳八寨创建国家5A级景区扎实推进，融水、三江与广西旅游发展集团合作开发旅游项目，创建特色旅游名县。知青城、都乐岩、龙女沟景区被评为国家4A级旅游景区，全市国家4A级景区达19家，位居全区第二。努力优化金融生态环境，银行业本外币存、贷款余额分别为2565.7亿元和1786.8亿元，分别增长9.0%和9.2%，政府性投资项目融资到位417.8亿元；柳州医药成为我市首家在A股市场上市的民营企业，天涌科技、爱格富食品科技、升禾环保在“新三板”挂牌，成功发行西部第一支增信集合债券10亿元。

　　夯基础促增效，加快农业现代化步伐。推进农业结构调整，新扩种水果12.7万亩，新种、改种桑树优良品种1.5万亩，粮食生产稳定增长，茶叶产量保持全区首位。养殖业规模化、标准化稳步推进，克服禽流感疫情及猪价下跌等不利因素影响，猪、禽产业仍然保持平稳增长。柳江县下伦双季莲藕示范区初步通过自治区现代特色农业（核心）示范区验收，“双高”糖料蔗基地启动建设，林下种养、“稻田养鱼”扩面增量。落久水利枢纽前期工作基本完成，洋溪水利枢纽建设方案通过广西贵州两省区论证，古偿河水库主体工程实现截流，导流隧洞通水。全市农村公路完成103个建制村道路硬化，通畅率达到80.9%。深入推进“农民人均纯收入倍增计划”，农业观光旅游、农产品商贸流通等措施有力促进农业增效、农民增收。全年实现农林牧渔业总产值277.7亿元，增长4.5%。

　　抓统筹促均衡，加快县域经济发展。柳江县力抓穿山工业园特色化建设，食品工业发展初见规模，百里柳江－凤凰河温泉景区（一期）建成运营。柳城县帮扶安琪酵母、鼎铭金属等重点企业发展，积极创建桑蚕特色农业（核心）示范区。鹿寨县强化与市区的交通及产业对接，加快推进热电联产项目、电镀工业园（一期）等重大项目建设。融安县建设广西香杉生态工业园（一期）工程，促进竹木深加工产业转型升级。融水县、三江县推进特色民族文化旅游业发展，积极打造“秀美融水·风情苗乡”和“千年侗寨·梦萦三江”旅游品牌，融水雨卜村、三江丹洲村、马安屯成为“广西特色旅游名村”。

　　（二）着力完善基础设施，扎实推进新型城镇化建设大力推进新区建设。新区发展框架进一步拉大，建成区面积24.57平方公里，比上年增加3.44平方公里。新区基础设施不断完善，花岭片、核心区、滨江区路网建设全面铺开，官塘污水处理工程厂区建设竣工验收，柳东大厦投入使用，华美达酒店开业运营，华侨城等一批项目开工建设。公共服务设施加快配套，景行小学柳东校区招生入学，鹿山学院（二期）、实验中学主体竣工。

　　大力拓展对外交通。积极推进铁路、公路、水运、航空等基础设施项目建设，完善城市综合交通规划修编，着力构建综合交通新体系。贵广铁路全线贯通，柳州火车站站前广场和湘桂铁路柳州至南宁段电气化改造工程加快推进，西鹅铁路货运中心站－东站还建项目开工建设，柳肇铁路（柳州至梧州段）项目由国家发改委批准立项。加快征地拆迁，积极推进柳州至梧州、三江至柳州、柳州至武宣、阳朔至鹿寨、桂林至三江等5条高速公路建设，柳南高速改扩建开始实施。

　　柳江航道疏浚和官塘作业区（一期）等港口码头建设如期推进。白莲机场改扩建工程开工建设，复航航线11条。

　　大力改善生态环境。生态建设持续加强，“绿满龙城”效果明显，完成植树造林面积33.4万亩，全市森林覆盖率达64.9%，城市建成区绿化覆盖率41.3%，城市人均公园绿地面积11.5平方米。柳江河饮用水保护河段继续保持国家地表水Ⅲ类以上水质标准，市区空气二氧化硫、二氧化氮均值达到国家二级标准要求。投资1.2亿元，进一步打造“生态花园·五彩画廊”，市区主要道路节点、游园绿地得到改造提升，“花园城市”效果更加彰显。

　　深入开展“美丽柳州·清洁乡村”活动，建成自治区“村收镇运县处理”试点乡镇18个、垃圾综合处理示范村屯120个、生态村84个，乡村环境卫生明显改观。划定85个乡镇饮用水水源保护区，建成28.9公里城镇污水处理管网，市区污水处理厂污泥集中处置工程、立冲沟生活垃圾填埋场渗滤液处理项目投入运行。

　　大力提升服务功能。加强和创新城市管理，率先在全区实施城市总体规划评估，充分发挥规划对优化城市空间、完善城市功能的引领作用。城市基础设施不断完善，南外环路、西南出口跨铁路立交桥等38项重大基础 设施项目建成使用，环江滨水大道、鹅山路东段改造等项目加快实施，古偿河输水管线和天然气长输管线加快建设，实施旧城区道路、排水和照明改造，城市承载能力不断提升。新建完成铁桥西等3个水上人工沙滩，为市民提供更多的滨水休闲空间。创建公交都市示范城市，BRT、水上公交开通运营后市内交通更加便捷；新增220个租赁点、4500辆公共租赁自行车，修建76.5公里慢行绿道，市民出行更加便利，河东片区步行和自行车交通系统改造工程入选“国家城市步行和自行车交通系统示范项目”；实施1023辆出租车油改气，有效减少汽车尾气污染。围绕“智慧城市”建设，重点实施公共信息平台、公共数据库、地下管网及“一卡多用，一卡通用”市民卡等工程。以管理促提升上水平，柳州连续六届获得“南珠杯”特等奖，鹧鸪江大桥荣获国家土木工程界工程技术最高荣誉“詹天佑奖”。严厉打击“两违”行为，全年依法拆除“两违”建筑面积147万平方米，城市建设更加规范有序。

　　（三）着力深化改革开放，不断增强发展内生动力深化各项领域改革。用好政府这只“看得见的手”，有序推进政府机构改革，加快转变职能，撤并整合文化广电和新闻出版、卫生和计生等部门，工作部门调整为40个，完成食品药品监管体制改革，增设133个市、县、乡三级监管机构，实现监管全覆盖。深化行政审批制度改革，加大简政放权，实施第八轮行政审批项目清理，继续取消减少119项审批事项，加快建立村（社区）级政务服务中心，覆盖率达到89%。深化国资国企改革，柳化集团资产重组、两面针公司定向非公开发行股票及柳州银行增资扩股等工作稳步推进，重点监管的40户企业资产增长20.3%，利润增长9.8%。推进公共资源交易改革，规范公共资源交易，市公共资源交易中心正式运转，完成交易项目1318宗，交易额113亿元。推进金融改革创新，促进金融良好发展，全市各类金融机构达76家，新型金融组织66家。加快发展普惠金融，积极推进小微企业信用体系（全国）试验区建设。加快农村金融改革，柳江县创建广西第一家农民自助“惠农通金融超市”，以点促面推动农村金融改革工作由试点转向全面实施。实施税收“营改增”改革试点，结构性减税效果明显，试点纳税人直接减税1.69亿元，激发了市场活力、增强了企业动力。工商登记制度改革进一步放宽经营场所登记，“先照后证”激发全民创业热情。全市新设立企业4427户，增长36.6%。实施促进非公经济发展“六大工程”，出台“1+5”文件，催生非公经济发展的活力与动力。农村综合改革以土地承包经营权确权登记颁证为重点，推动土地流转30.5万亩，惠及近5万农户。医疗卫生体制改革重点实施城市公立医院改革和县级公立医院综合改革，推行城乡居民大病保险。深化教育体制改革，完善多元办园的学前教育体制，破解“入园难”问题；在鹿寨、三江两县开展“县属轮教”试点，合理配置优质教育资源，促进教育均衡发展。

　　扩大对外开放合作。成功举办第四届中国－东盟（柳州）汽车、工程机械及零部件博览会，展会规模与专业化水平有新提升，深化了与国内外汽车及零部件企业的交流合作。开展中欧产业合作（柳州）投资洽谈会，优势互补、互利共赢，积极推进与欧洲各国企业的开放合作。支持有条件的企业“走出去”，对外投资快速增长，全年完成对外投资1.69亿美元，同比增长3.6倍。加强互联互通，推进区域合作，柳来河区域合作一体化取得新进展，与贵州省黔东南州签署协议全面推进战略合作。与香港各界青年开展“共和国之旅－共筑中国梦”互动，增进友谊，促进交流合作。创新招商引资方式，全程服务抓项目落地，引进市外境内到位资金（含续建项目）625亿元，增长19.4%；利用外资（全口径）实际到位资金3.7亿美元。

　　（四）着力保障改善民生，促进社会事业协调发展民生质量继续提高。社会保险覆盖范围不断扩大，参保总人次达358.2万。关注特殊和困难群体，完善“三无人员”居民医保政策，解决失地农民参保缴费难问题。在连续9年基础上继续上调基本养老保险待遇，使月人均养老金提高到1882元。统一市县失业保险待遇支付标准，县级失业人员失业保险金平均上涨44%。实行居民大病保险即时结算，4.93万人次享受大病保险待遇。保障性住房建设全区领先，实行公租房和廉租房并轨运行管理，实物建房开工13459套，基本建成16012套，分配入住8729套。

　　完成农村危房改造11441户、城乡风 貌改造2184户。实施农村饮水安全工程413处，受益群众18.6万人。扩大学生免费午餐受益面，7所特教学校485名学生纳入免费午餐工程，全市受惠学生达18.33万人。给全市80－89岁老人每月发放50元生活补贴，将90岁以上老人生活补贴提高到每月100元。关爱计划生育“失独家庭”，给“失独夫妻”每人每月发放500元扶助金。为230名贫困截肢残疾人免费安装假肢。实施精准扶贫，全市农村减少贫困人口5.1万人。

　　社会事业不断加强。创建国家卫生城市通过综合评审，历史文化名城保护整改工作通过国家有关部门验收。

　　柳州工业博物馆“生态宜居馆”建成开放，“村村通”广播电视乡镇无线覆盖工程提前完成，《八桂大歌》升级版成功展演，《弦舞蜻蜓之蜻蜓坊》赴北京水立方等地演出，“鱼峰歌圩”被文化部授予“中国民间文化艺术之乡”，柳州艺术馆成为我市陈列展览名家书画、摄影等各类艺术精品的重要平台。积极开展全民健身活动，市区及六县公共体育场馆免费或低收费对社会开放，群众体育锻炼蔚然成风。

　　竞技体育成果丰硕，柳籍运动员韦永丽等在第十七届亚运会上创造佳绩，柳州代表队在广西第十三届少数民族运动会、广西第八届残疾人运动会上取得优异成绩。坚持群众参与、形式多样，2014年水上狂欢节、第八届奇石节办得精彩纷呈、更具特色，吸引各地游客纷至沓来观光旅游。义务教育均衡发展学校基本建设三年攻坚计划加快推进，文华中学、古亭山小学等7所新建（迁建）学校正式启用。

　　优化职业院校办学资源，组建广西汽车产业职教集团，柳州铁道职院和城市职院迁入职教园区，办学条件进一步改善。基本药物制度实施范围进一步扩大，全面完成五年防艾攻坚工程，柳铁中心医院1号住院大楼投入使用，中医院东院和多个县级卫生项目开工建设。人口计生工作健康发展，“单独两孩”政策稳步实施。深入推进“平安柳州”建设，社会治理网格化服务管理全面覆盖市区及县城，综合信息化管理延伸到乡镇；实施“天网”工程五期，进一步完善立体化、现代化社会治安防控体系；加强校园内外及重点地区治安环境综合整治，社会治安保持平稳，公众安全感持续提升。

　　扩大法制宣传和法律援助覆盖面，建成法治文化公园。食品药品安全专项整治取得成效，强化群众饮食用药安全保障。建立隐患排查治理体系，加强安全生产监督与管理。深入开展全国文明城市创建活动，大力加强国防后备力量建设，扎实推进军民融合双拥共建，继续创建全国双拥模范城，努力实现“八连冠”。民族团结进步事业不断发展，社会主义核心价值观和“中国梦”深入人心。

　　（五）着力转变工作作风，提高政府行政效能扎实开展第二批党的群众路线教育实践活动，聚焦“四风”，剖析查摆问题，认真开展批评和自我批评，不断改进工作作风，倡俭治奢、倡勤治懒，进一步密切同人民群众的血肉联系，大力推进为民务实清廉政府建设。

　　严格落实中央八项规定，大幅减少市级会议及文件，压缩“三公”经费7100万元，同比减少23.9%。调整多占办公用房1.39万平方米，停建楼堂馆所12个。严格落实“财政供养的人员只减不增”有关要求，机构编制得到有效控制。深入开展领导干部接访下访、市长“企业接待日”、“千名干部入千企”对口服务、“中小企业服务月”等活动，努力化解信访积案，切实做好与群众利益密切相关的实事好事，积极解决联系和服务群众“最后一公里”问题。认真执行市人大及其常委会的决议、决定，自觉接受人大依法监督、政协民主监督，主动听取各民主党派、工商联和社会各界意见，全年办理人大代表建议132件、政协委员提案254件，办结率均为100%、满意率均达99%以上。深入推进廉政建设，严格落实党风廉政建设责任制，严肃查处违法违纪案件。加强“12345”政府热线服务，不断扩大覆盖面，受理群众投诉9.79万件，办结率98%，网络在线问政、行风在线评议等互动平台更好发挥联系群众服务群众的作用。

　　过去一年，我市外事、侨务、统计、审计、粮食、技术监督、国家安全、人防、气象、防汛抗旱、防震减灾、妇女儿童、宗教、老龄、决策咨询、档案、地方志、哲学社会科学等各项工作都取得了新的成绩。

　　各位代表，过去的一年，我们取得的成绩来之不易，这是市委总揽全局、科学决策的结果，是市人大、市政协监督支持的结果，是全市人民团结拼搏、开拓进取的结果，是社会各界鼎力支持、积极奉献的结果。在此，我代表市人民政府，向全市人民，向驻柳人民解放军、武警部队官兵，向 各民主党派、工商联、人民团体及海内外所有关心和支持我市发展的各界人士，致以崇高的敬意和衷心的感谢！在总结成绩的同时，我们也清醒地认识到存在的困难与问题，主要是：经济下行压力仍然较大，产业结构性矛盾还比较突出，部分传统产业优势有弱化趋势，战略性新兴产业尚未发展壮大；实体经济特别是中小企业融资难、融资贵问题突出，部分企业经营困难；生产要素综合成本上升、节能减排等环境约束加强；城乡区域发展不够均衡，农民增收渠道不多，部分群众生活还比较困难，等等。对于这些困难和问题，我们要敢于面对、勇于担当，采取有效措施，认真加以解决，把全市改革发展各项事业继续推向前进。

　　二、2015年工作总体要求和目标任务

　　2015年是全面深化改革的关键之年，是全面推进依法治国的开局之年，也是全面完成“十二五”规划的收官之年。当前，国内外经济发展不确定因素依然较多，世界经济仍处在国际金融危机后的深度调整期，世界经济增速可能会略有回升，但总体复苏疲弱态势难有明显改观。我国经济发展进入新常态，正从高速增长转向中高速增长，经济发展方式正从规模速度型粗放增长转向质量效率型集约增长，经济结构正从增量扩能为主转向调整存量、做优增量并存的深度调整，经济发展动力正从传统增长点转向新的增长点。新常态下我们面临一些新问题、新挑战，但也迎来诸多新机遇、新发展。国家实施“一带一路”等三大战略，广西贯彻落实珠江－西江经济带发展规划、大力实施“双核驱动”，积极加快西江经济带建设，新一轮发展布局全面展开，新一轮竞争格局逐步形成。要认真总结“十二五”规划实施情况，深入研究国家、自治区相关政策，选择对接国家和自治区战略规划的切入点，紧紧围绕事关柳州的全局性重大问题，开展研究论证和前期工作，明确定位、选准目标，精心编制好“十三五”规划，力争一批重大基础设施工程、重大产业项目、重大民生项目纳入国家、自治区“十三五”规划盘子，使柳州在全新的发展大局中实现更大作为。要自觉认识新常态、主动适应新常态，顺应国际国内经济发展大势，把握柳州发展的阶段性、结构性特征，锐意进取，主动作为，统筹推进稳增长、调结构、促改革、谋发展各项措施，努力开创新常态下柳州改革发展新局面！今年政府工作的总体要求是：全面贯彻落实党的十八大、十八届三中、四中全会和中央、自治区经济工作会议以及市十一届党代会第二次会议精神，大力实施“实业兴市，开放强柳”战略，牢牢把握稳中求进的工作总基调，主动适应新常态，全面深化改革，全面推进法治建设，坚持总量提质、分量提速，提高经济发展质量和效益，坚持大力改善民生、发展社会事业，加快经济升级、城市转型，奋力实现“十二五”规划目标，努力打造西江经济带核心城市，建设“五美五好”柳州。

　　今年我市经济社会发展的主要预期目标是：地区生产总值增长8.5%；工业总产值增长10%；固定资产投资增长16%；财政收入增长8%；社会消费品零售总额增长12%；城镇居民人均可支配收入增长10%；农民人均纯收入增长11%；单位生产总值能耗、主要污染物排放总量控制在自治区下达指标以内；城镇登记失业率控制在4.5%以内；人口自然增长率控制在8.5‰以内。

　　做好今年的工作，要着重把握以下几个方面：

一是主动适应新常态，努力保持经济持续稳定增长。经济发展新常态下，我们要充分把握经济发展新规律新特点，结合柳州新型工业化、城镇化、信息化、农业现代化的发展现状，主动谋划，顺势而为，牢牢把握发展主动权。当前的柳州，尽管经济下行压力较大，但经济发展长期向好的基本面没有变，经济持续健康发展的态势没有变。要按照全区经济工作会议所提出的那样，在有质量有效益的前提下发展得更快一些。要努力促进经济发展总量、质量、分量“三量齐升”，保持经济持续稳定较快增长。

　　现阶段工业还是推动柳州经济增长的主要支撑，投资和消费仍是稳增长的主要动力，城镇化将是释放发展潜力的主要空间，现代服务业则是产业结构调整的主攻方向，我们既要加快经济发展，又要保持生态环境，实现强市富民和生态文明有机统一；既要扩大投资加快项目建设，又要创新筹融资模式提高投资效益，实现又好又快发展；既要提高行政效率能快就不要 慢，又要依法行政加快法治政府建设，努力在经济发展新常态下实现更大作为。

　　二是牢牢把握发展新机遇，努力打造西江经济带核心城市。全面贯彻落实珠江－西江经济带发展规划，围绕交通、产业、生态、城镇化、开放合作、公共服务六大建设重点，在建设转型升级的现代产业经济带、经脉畅通的综合交通运输大通道、宜居宜业的特色沿江城镇带和千里生态廊道中，找准柳州定位，积极争取中央和自治区相关政策支持，加快推进规划中涉及柳州的重点项目和重大工程建设。

　　以实业支撑发展，以产业集群化助推形成区域分工协作体系，加快打造区域性先进制造业基地，积极发挥集聚效应；以柳肇铁路、柳韶铁路等建设为重点，以打造2000吨级航道为突破，构建交通运输新优势，加快打造区域性现代服务业中心，积极发挥纽带效应；扎实推进低碳经济、节能减排、循环发展，深入开展空气、水和环境综合治理，促进转型升级和生态文明建设，实现“生产、生活、生态”共赢发展，积极发挥示范效应，努力打造西江经济带核心城市。

　　三是全面深化改革开放，努力增强经济社会发展动力。按照中央、自治区决策部署，认真谋划既具有年度特点、又有利于长远制度安排的改革举措。从制约我市经济社会发展的瓶颈问题和群众反映突出的热点难点问题改起，重点推进投融资、行政审批、土地管理、财税金融等领域改革，使改革举措有效转化成发展动力。准确把握对外开放出现的新特征，大胆探索、先行先试，积极构建开放型经济新体制，努力在体制机制和市场体系方面同发达地区接轨。主动适应区域合作大趋势，抓住多重叠加机遇，充分借助中国－东盟博览会等平台，更加积极主动融入珠江－西江经济带和北部湾开发建设，不断拓展与泛珠三角、西南中南地区及东盟国家的交流合作，加快柳来河一体化进程，在全新的发展格局中实现互利共赢。

　　四是更加注重民生，努力保持社会繁荣稳定。在经济换挡减速的新常态下，推进民生发展要有“新状态”，更要有“新作为”，要像抓经济建设一样抓民生保障，像落实发展指标一样落实民生任务，坚守底线、突出重点、完善制度、精准发力，多些雪中送炭，更加注重改善基本民生、保障底线民生、抓好热点民生，更加关注低收入群众、特殊困难群体和大多数群众利益。对教育、扶贫、就业、社会保障、卫生医疗、食品药品监管等老百姓关心、关注、期盼的问题，要加大投入、真下功夫、狠抓落实，一件接着一件办，一年接着一年干，锲而不舍往前推，让柳州人民生活更加美好。

　　五是服务发展主题，努力建设法治政府。依法全面履行政府职能，健全依法决策机制，深化行政执法体制改革，严格规范公正文明执法，强化对行政权力的制约监督，全面推进政务公开。围绕基本建成法治政府的目标，结合柳州实际研究制定符合中央要求、体现人民意愿的具体措施，狠抓工作落实，着力推动各级政府及部门工作人员树立法治理念、增强法治思维，运用法治方式，推动发展、化解矛盾、维护稳定，不断提高依法行政、依法治市的能力和水平。

　　三、2015年工作安排

　　今年，我们要全力抓好以下几个方面的工作：

（一）围绕工业提质增效，盘存量优增量促升级把做大工业总量、提升发展的质量和效益放在突出位置，调整生产方式和管理模式，推动新技术、新产业、新业态、新模式发展，形成产品高端化、制造智能化、产业绿色化、发展集约化，促进工业加快转型升级。

　　加快转方式调结构促升级。以产业升级、产品结构优化为重点，加快推进企业技术进步和自主创新，提升市场竞争能力。汽车产业围绕规模质量再上新台阶新水平，巩固微型车优势，重点发展高附加值轿车、MPV、SUV新产品，上汽通用五菱力争产量突破200万辆、产值达到850亿元，汽车工业争取实现产值增长13%以上。

　　钢铁产业瞄准市场加快技术进步，生产品种钢等更多适销对路产品，柳钢力争实现钢材产量1500万吨、产值700亿元。机械产业加大智能化、集成化、机电仪一体化改造，提升核心零部件和新产品研发制造能力。其他传统产业也要紧紧抓住当前大宗原燃料价格下跌并处于低位的时机，围绕市场需求调整产品结构，提高企业效益。

　　产业结构优化升级需要以项目为支撑，要加快百项工业结构调整项目建设，年内竣工投产欧维姆洛维生产基地、新宇纺织“20万锭纺纱能力提升改造”等50多个工业项目，尽快形成新的工业经济增长点，抓紧推进柳州卷烟厂百万箱技术改造等续建项目，开工建设柳钢矿渣微粉生产线、正堂药业搬迁改造等一批新项目。要围绕完成更新改造投资1000亿元，做好资金筹措、保障土地供应、抓好征地拆迁，扎扎实实推进项目建设。

　　大力发展战略性新兴产业。推动战略性新兴产业加快发展，有利于形成区域经济技术合作和竞争发展的新优势，要坚持以创新引领，以新能源汽车、智能制造装备、生物医药、电子信息和节能环保等领域为突破口，在市场准入、土地使用、财政支持和金融服务等方面创造宽松的发展环境，给予优惠的扶持政策。围绕“引进一个领军人才，发展一个高科技企业，带动一个新兴产业成长”，支持企业引进高层次创新人才。推动上汽通用五菱新能源汽车、东风柳汽混合动力汽车研发生产，实施年产3万吨新型特种钢高强度焊丝、安全防控智能电子产品等新兴产业项目。筛选出一批市场前景好、自主创新能力强的企业加以重点扶持，不断做强做优做大。推动战略性新兴产业产值突破400亿元，占全市比重达到10%以上。

　　坚持创新驱动。把增强企业自主创新能力作为转变经济发展方式的重要支撑，坚持“一企一策”，推动一批企业加强技术创新、商业模式创新、品牌创新，以市场和客户需求为导向，开发新产品，改进服务模式、管理模式、营销模式，成为创新驱动发展的生力军。要完善创新能力体系建设，促进工业化和信息化深度融合，投资28亿元实施130项技术创新项目，新培育20家左右自治区级和市级企业技术中心，启动创建国家级创新型试点城市、自主创新示范区和新能源汽车推广应用示范城市。深入实施质量强市战略，争创“全国质量强市示范城市”，加快推进国家汽车质量监督检验中心（柳州）、国家进出口汽车检测重点实验室（柳州）等创新平台建设，紧紧依靠创新驱动培育和形成更多新的经济增长动力。

　　打造特色工业园区。围绕产业聚集发展，明确园区产业定位，推动园区特色发展、错位发展。围绕高端化、配套化、集聚化，大力引进带动性强的龙头项目，打造一批产业特色突出、创新能力和配套条件好的高端产业集群。着力推动汽车城建设提速增效，重点实施上汽通用五菱宝骏基地（二期）、东风柳汽乘用车基地（二期）等项目建设，竣工投产上汽通用五菱涡轮增压发动机、玲珑轮胎1000万套高性能轮胎等项目。同步建设汽车城零部件配套体系，加快联合电子、福耀玻璃等项目建设，进一步完善汽车配套全产业链。围绕打造创新型特色园区，重点推进电子信息产业园、中欧产业园、穿山产业园、纺织产业园等特色产业园建设，与牛津大学合作共建柳州国际高科技园，推进河西工业园申报自治区级高新技术开发区，力争阳和工业新区“全国专用车零部件产业知名品牌创建示范区”通过验收。

　　完善园区基础设施，针对各工业园区不同的功能定位，因园施策，重点抓好园区配套供水供电、污水处理、公共服务等方面建设，不断提升园区承载能力、服务功能和水平，促进园区产业加快发展。全市工业园区工业产值增长10%以上。

　　（二）加大“三农”工作力度，促进农业增效 、农民增收 、农村发展加快转变农业发展方式，大力发展现代农业，使之与工业化、城镇化和信息化的发展相协调，积极发展多种形式、适度规模经营，让农民从产业链增值中获得更多利益。

　　着力发展现代农业。推进农村土地确权和流转，支持农民合作社、家庭农场、农业企业等新型经营主体发展，积极发展农产品流通企业、农产品运销大户等市场主体，加快推进农业现代化。抓紧实施11个现代特色农业（核心）示范区建设，充分发挥引领带动作用。进一步优化农业产业结构，扩大晚熟柑桔、蜜橙、滑皮金桔、双季葡萄等特色水果种植面积15万亩，扩改种桑园2万亩，继续推进“双高”糖料蔗基地建设，打造一批高标准蔬菜园区，培育蔬菜、水果、畜牧水产等深加工产业。深入实施农产品质量安全工程，强化农业标准化生产，建立健全农产品质量安全追溯体系。实施“南菜北运”“农超对接”，启动“车载农产品生鲜进社区”，健全完善农产品流通体系。推进农机化创新示范工程，提升农业机械化水平。

　　改善基础设施条件。重点围绕改善水利设施、道路交通等基本状况，加快推进一批项目。开工建设落久水利枢纽工程，做好洋溪水利枢纽工程前期工作，完成古偿河水库大坝混凝土工程。编制《柳州市新型城镇化城镇（群）发展建设规划》，实现各县城控规全覆盖，以规划指引推进各方面建设。启动国道322柳江绕城公路改线工程，全线贯通柳东大道延长线，加快实施沙塘至沙埔道路改造、罗城至洞头公路融水段道路等工程。完成一批建制村农村公路项目，努力实现全市通村道路通畅率达100%。推进农业基地水利“最后一公里”工程，建成一批水果、蔬菜、桑蚕、茶叶等水肥一体化项目。

　　持续提高农民收入。培育产业、搞活流通、发展经营，推进倍增计划十大产业工程、十大增收行动，促进农民获利增收。建设一批精品果园、优质菜地、高效桑园、标准茶园及“万元养殖小区（村、场）”和林业经济基地。新增5个“万元示范村”、105家“十万元示范农户”，发展农村个体私营工商经营户1000家，农业观光旅游村（园）达到35个、农家乐达到800家，新增农村劳动力转移就业5万人。推行各类土地使用权、经营权及地上设施抵押贷款试点，发挥土地资源的资金效益，增加农民财产收益。

　　深入推进精准扶贫，用好用足国家、自治区扶持贫困县发展政策，帮助贫困群众脱贫增收。

　　激发县域经济活力。营造有利于各类县域经济主体创新创业的环境，催生壮大县域经济发展内生动力。各县要因地制宜、突出特色、科学定位、差异化发展，柳江县重点打造汽车及零部件、机械制造、食品产业等特色产业集中区，加快推进新兴工业园产城融合；柳城县突出发展生物医药、新材料等新兴产业，着力推进特色农产品产业化；鹿寨县加快建设国家循环化改造示范试点园区和桂中现代科技林业产业园、电镀工业园，积极打造鹿雒经济带；北部三县可发展风电、制药、竹木深加工等生态型工业，以及茶叶、油茶、滑皮金桔和“稻田养鱼”等特色农业，融安县还可打造广西香杉生态工业基地，延伸香杉产业发展链条，融水县和三江县还可加快旅游基础设施建设，创建民族风情和特色旅游品牌。

　　（三）加快提速增量，大力发展现代服务业坚持现代服务业与传统服务业并重，生产性服务业与生活性服务业并举，促进现代服务业与工农业协同发展。着力发展现代物流、电子商务、互联网金融等新业态、新模式，推动现代服务业发展壮大。

　　繁荣城市商圈。合理优化商业布局，形成功能特色各异的城市商圈，不断提升城市商业品质。加快推进万达、华润、恒大等服务业重点项目建设，推动秀品天地等商业综合体开业营运，发挥集聚效应，打造河东新商圈；做好地王国际财富中心等项目，优化商务中介、总部经济、信息咨询等业态配置，助推河北商圈提升换档；引导新时代商业港、飞鹅批发市场创新商业模式，促进河南商圈转型升级；竣工新柳邕农产品批发市场（一期）并投入运营，完善壮大河西商圈。

　　加快发展现代物流业。适应产业发展的需求，积极构建大进大出的生产流通格局，打造大型产业物流集聚区，加快建设汽车、钢铁生产性物流基地，创建汽车城生产性服务业示范区。建设鹧鸪江钢铁物流园（二期）、柳州国家公路货运枢纽柳东物流中心、桂中海迅柳北物流基地（二期）、长乐物流园等一批重点物流项目。依托区位、产业、交通优势与信息化发展，规划建设空港物流园区。

　　大力发展电子商务。发展电子商务等新业态，既能形成新的经济增长点，又能促进服务业优化升级。要把电子商务作为现代服务业发展的重点，制定促进电子商务产业发展的政策措施，实施“宽带柳州”“电商柳州”工程，创建国家电子商务示范城市。

　　着力打造电子商务平台，推进阿里巴巴“柳州产业带”和淘宝“柳州特色馆”上线运营，积极引进大型电子商务、互联网和快递物流企业，促进高新技术开发区、新时代商业港等电商园区或电商基地建设。

　　推进旅游名城建设。把旅游业作为重点发展的产业，作为服务业需要加力补齐的短板，科学合理开发旅游资源，精心打造旅游精品景观。积极创建百里柳江、三江程阳八寨国家5A级景区，完善三江侗族风情景区、融水元宝山－贝江景区旅游配套设施，创建三江、融水广西特色旅游名县。提升鹿寨香桥等旅游景区开发水平，推出窑埠古镇等新景点。抓好乡村建设规划，突出农业产业带动，打造一批生态好有特色的示范村，促进城郊型农业观光旅游发展。以贵广高铁开通为契机，加强与沿线城市开展旅游合作，加快融入粤桂黔高铁旅游经济圈。

　　（四）深化改革创新，激发活力增强动力以问题为导向，全面推进重要领域、重点环节改革，破除体制机制弊端，破解发展瓶颈，释放更大的改革 红利，助推经济社会又好又快发展。

　　深化经济领域改革。按照中央和自治区的部署要求，稳妥有序推进各项改革。深化投融资改革，深入研究《国务院关于加强地方政府性债务管理的意见》（国发[2014]43号），推进投资主体多元化，增强对社会资本的吸纳、带动力，推动创业投资和股权投资加快发展，大力发展市场直接融资，为项目建设提供资金保障。深化国资国企改革，进一步理顺产权关系，强化出资人管理，建立以管资本为主、运行高效的国有资产监督管理体制，做到监管科学、规范有序，提升国有企业竞争活力。深化预算管理制度改革，依法组织财政收入，严格预算支出管理，积极推进预算公开，集中财力优先支持重点园区、重点产业、重点项目，支持教育、扶贫、社会保障等重大民生事业。积极推进“营改增”和消费税等税收改革，激励市场主体，推动产业结构、需求结构和就业结构不断优化。深化农村综合改革，统筹实施农村“六权”确权登记颁证，推动农村土地规范有序流转，加快农村金融改革，推进农业财政贴息贷款和政策性农业保险。改革创新金融服务，促进多层次资本市场发展，加强区域集优直接债务融资和新型金融组织监管，积极做好柳州银行、欧维姆等上市准备，推动企业IPO上市及“新三板”等场外交易市场挂牌；深入开展小额贷款保证保险试点，设立创业投资引导基金和企业周转基金，完善融资担保体系，加快建立企业信用评价系统，改善中小企业融资环境。改革创新服务方式，大力发展非公有制经济，进一步降低非公经济市场准入门槛，推动非公有制企业改制上市、债券融资、科技创新，迸发出更大经济活力。

　　深化行政和社会管理改革。深入推进行政审批制度改革，进一步清理下放行政审批事项，完善行政审批事项目录管理，制定公布权力清单、责任清单、负面清单和财政专项资金管理清单。全面实施工商登记“先照后证”，加强事中、事后监管，激发市场活力和创造力。按照中央和自治区的要求，严控事业编制总量只减不增，推进公务用车制度改革，完成市县不动产登记职责整合与机构设置。推进公共资源交易体制改革，推行公共资源交易电子招投标，建设完善统一规范的交易平台。推进教育领域改革，促进教育均衡化发展，完善县域内教师轮岗交流机制，支持社会力量办学，加大对民办幼儿园的扶持。深入推进医药卫生体制改革，完善城市公立医院改革配套政策，加快推进县级公立医院改革，实行基层医疗机构门诊总额预付。深化文化体制改革，以市博物馆、鱼峰区为试点探索建立公共文化机构法人治理结构，努力构建充满活力、适应人民群众对文化生活新需求的体制机制，进一步激发文化活力。

　　（五）搭建开放合作新平台，拓展发展新空间主动融入国际交流合作、区域产业协作，在更大范围集聚发展资源，优势互补，合作共赢，努力打造内陆城市开放新高地。

　　深化区域经济合作。主动融入珠江－西江经济带联动开发，积极参与区域产业分工合作与交流，加快实施公路、航运、生态保护、加工贸易等合作项目，不断凝聚区域经济发展合力。

　　加强与北部湾经济区各城市的合作，重点在交通、能源等基础设施方面谋划和建设项目，拓展资源配置空间。

　　进一步扩大与东盟全方位合作，深化与西南中南地区合作交流，加强与贵广高铁沿线地区的合作开发，共同打造贵广高铁经济带，努力在先进制造业、战略性新兴产业等方面创立新合作平台，在资源开发、商贸物流、文化旅游等领域拓展新发展空间。

　　大力实施“走出去”战略。加强国家汽车及零部件出口基地建设，搭建公共服务平台，完善出口汽车及零部件质量安全示范区、工程机械产品售后服务平台建设，提高出口产品竞争优势和效益。加快推进官塘综合保税区申报工作，统筹推进口岸陆运转关、航空口岸、码头作业区建设，促进便捷通关。加快贸易方式调整，依托产业优势，扩大汽车、工程机械等优势产品出口，实施“加工贸易倍增计划”，优化外贸出口结构。支持上汽通用五菱在印尼独资建厂，推动柳工、欧维姆扩大海外投资，不断提高柳州产品国外市场份额。

　　全面提升招商引资质量。进一步完善招商引资信息共享协调机制，统筹推进市县、市区联动招商。加强高新区、开发区、特色工业园区产业招商，引导内外资更多投向战略性新兴产业和先进制造业，提高产业集中度和产出率。创新招商项目评审、推进、问责机制，注重聚商选资，提高招商实效，努力引进青年客车、芭蕾雨奥特莱斯等一批带动力强的好项目大项目。坚持市场化运作办好第五届中国－

　　东盟（柳州）汽车、工程机械及零部件博览会等展会，吸引更多客商走进柳州、投资柳州。争取全市引进市外境内资金增长8%，利用外资（全口径）增长19%。

　　（六）秉承绿色发展，打造现代生态都市坚持生态优先、绿色发展，把生态文明建设放在更加突出位置，不断强化环境治理与生态保护，让柳州青山常在、绿水常流，现代文明与生态文明交相辉映。

　　建设宜居宜业现代新城。以柳东新区为新型城镇化发展的战略承载地，坚持规划引领，突出能级提升，全力打造产城融合、功能配套、环境优美、充满活力的现代新城。加快推进CBD核心区和新区公共服务配套设施建设，进一步完善新区滨江大道、火炬大道等路网，开工建设柳东新区水厂（一期）和天然气利用等工程，竣工儿童医院（一期）以及中小学校等项目，优化供水、供气、供电、排污等管网设施，提升新区综合承载功能。拓展城市发展新空间，科学规划沙塘、东泉、柳城华侨农场等市区北部区域，围绕承载高端制造业及绿色工业、创意研发、科教文化、生态休闲体验等产业发展，开展“沙东（沙塘－东泉）生态科教新城”规划研究。

　　打造提升综合交通枢纽体系。抓住自治区推进“市市通高铁”“县县通高速”的机遇，开工建设柳肇铁路（柳州至梧州段）工程，推进柳州东站还建、柳州站站房扩建等项目，实施衡阳至柳州段、怀化至柳州段电气化改造等项目前期工作。竣工阳朔至鹿寨、柳州至武宣高速公路，加快桂林至三江、柳州至梧州、三江至柳州、柳南改扩建等高速公路建设。抓紧实施柳州江口作业区（一期）、官塘作业区（一期）等工程，启动红花枢纽二线船闸建设。加快推进柳州机场航站楼及配套设施扩建工程，谋划建设空港经济区。

　　完善城市基础设施。进一步优化城市路网交通，竣工桂中大道延长线、学院路延长线、环江滨水大道等工程，实施河东南片区路网改造、城邕路、鹅山路东段下穿通道等项目。深入开展公交都市创建，竣工河东综合交通枢纽中心和汽车南站改造工程。继续推进慢行交通系统、步行和自行车交通系统改造，新建公共租赁自行车（三期）项目。进一步优化联接新区与旧城的交通网络系统，启动城市轨道交通前期工作。大力推进市政及公共服务设施建设，实施古偿河第二水源工程、中缅天然气市区配套及管线工程等重大工程项目。加快静兰片区升级改造和白沙村、帽合村、水南村等城中村综合改造，改善城市面貌，不断提升城市品位。

　　加强生态环境建设。积极探索和建立工业城市生态文明建设长效机制，努力打造“中国工业生态文明城”。加强城市环境综合整治，开展城市卫生和市容市貌百日整治大会战。深入创建国家环保模范城市，扎实做好重金属、大气和水体污染防治，进一步扩大污水管网覆盖率，开展雨水收集利用建设试点，提高生活废水及垃圾的回收、集中处理水平。大力发展绿色低碳循环经济，促进产业发展生态化、生态经济产业化，扶持一批清洁生产、循环利用、零排放企业发展壮大，实施柳钢原燃料场封闭工程，尽快启动柳化、柳州电厂、新兴糖厂退城进园升级改造，推进鹿寨循环化改造及热电联产项目。做好水源林保护，利用汛期加大河流冲刷，提升自我净化能力，减少沉积改善水质，保持柳江流域良好的生态环境。实施“绿满龙城”“家园绿美”工程，不断丰富“生态花园·五彩画廊”花园城市内涵。深入开展“美丽柳州·生态乡村”建设，绿化村屯400个，分类建设产业带动、有民族民居特色、格调鲜明的美丽乡村。

　　提升精细化管理水平。坚持市容整洁、景观优美、管理规范、市民满意，不断谋划新思路新举措，进一步加强城市管理。强化城市环境卫生管理，进一步做好道路保洁，规范垃圾清运处置，狠抓渣土扬尘污染整治。

　　开展市容市貌专项整治，以市区主要道路为载体，集中整治户外广告、流动摊担、店外经营等突出问题，抓好重点地区周边和重点路口综合整治，以打击“两违”的高压态势，遏制违法建设、乱搭乱建。提升市政建设维护水平，积极推进道路路面维护、城市排水管道疏浚等工作，确保基础设施完好、使用功能完善。提高管理效能，加强数字化城管信息平台建设，推进政务、民生、基础设施等领域智能化应用，完善管理机制，提高整体联动、应急处置能力，适应城市化快速发展的管理要求。

　　（七）坚持民生为先，增进人民福祉秉持以人为本，坚持以民为先、民生为大，帮群众所需，解群众所难， 让柳州百姓生活得更加幸福美满。

　　增强社会保障能力。加大社会保险扩面征缴力度，稳步提高各项社会保障待遇标准，让广大人民群众更多受益、更好受益。加快推进养老服务体系建设，提高特困对象（包括城乡重度残疾人、贫困残疾人、城镇“三无”人员、农村五保供养对象）养老金标准。发展社会福利和慈善事业，做好优抚安置工作，完善残疾人社会保障和服务，不断提高社会救助服务水平。推进新农合市级统筹试点，优化农村医疗救助经费即时结算管理平台，探索异地就医结算。切实保障农村留守老人、留守儿童、留守妇女权益，提供更多就业服务，努力协调帮助一些夫妻双方外出务工的困难家庭实现一方近家就地务工，以便照顾孩子及家庭。进一步完善农村公共服务体系，改善农村人居环境，提高农民素质，建设“人的新农村”。

　　抓好民生工程建设。继续实施10项为民办实事项目，在市区主要公共场所开通免费无线网络（WIFI服务，预测预报未来72小时空气质量情况，通过政府贴息、银行提供优惠利率贷款的方式，帮助中断缴费的困难人员续缴城镇企业职工基本养老保险，着力解决老百姓普遍关心的热点难点问题。不断满足群众基本住房需求，加快推进职工危旧房、城中村、老旧住宅区等安居工程项目，计划开工实物建房14920套，基本建成12000套，力争城镇居民住房保障覆盖面达到21%。加快农村地区危房改造、道路硬化、垃圾处理、生态建设，改善生产生活条件，积极帮助贫困村整村脱贫和贫困户持续增收，完成“十二五”确定的183个贫困村整村脱贫工作，齐头并进建设“物的新农村”。

　　切实增强社会治理能力。深化“平安柳州”建设，完善立体化治安防控体系，全面推进农村综治信息化、管理网格化，抓好禁毒工作，有效遏制吸毒打击制毒贩毒，最大限度消除毒品危害，严厉打击各类违法犯罪活动，全面提升人民群众安全感。进一步完善法律服务机制，提高法律援助覆盖面，妥善解决群众“公证难”问题。提高信访事项办理效能，及时回应群众合理诉求，依法化解疑难信访积案和涉法涉诉信访案件，严格执行信访结案制。始终把安全生产作为一切工作的“底线”，进一步强化安全意识，健全安全生产责任体系。严格落实政府部门监管责任，“谁主管，谁负责”；严格落实县区、乡镇属地管理责任，守土有责、守土负责、守土尽责；依法强化企业安全生产主体责任，切实落实各项安全措施，坚决遏制重特大安全事故。建立覆盖全过程的食品药品监管制度，治理餐桌上的污染，守护“舌尖上的安全”。积极完善防灾减灾救灾、突发事件应急管理体系，及时妥善处理各类突发事件，营造和谐稳定的社会环境。

　　（八）繁荣社会事业，更好服务群众顺应人民群众对文化、教育等公共服务的新需求，努力打造文化精品，不断提升教育质量，加快改善医疗卫生条件，促进各项社会事业健康发展，为群众提供更多更好服务。

　　鼓励大众创业万众创新。实施“个十百人才工程”“优秀人才接触柳州计划”等就业促进计划，创造提供更多的就业岗位，力争年内新增城镇就业5万人。营造有利于促进创业的发展环境，激励和扶持科技人员、大中专毕业生、返乡农民工等各类创业群体自主创新创业。积极搭建创新创业平台，加强高新技术创业服务中心、留学生创业园、信息产业园等平台建设。深化政产学研合作，强化科技创新对实业发展的支撑。

　　优先发展教育事业。围绕“学前教育扩规模、促普惠，义务教育抓标准、促均衡，普通高中抓质量、扩优势，职业教育抓品牌、促内涵”加强软硬件建设，努力办好人民满意的教育。继续推进学前教育三年行动计划、义务教育均衡发展三年攻坚计划，实现学前教育三年毛入学率达到76%，义务教育巩固率提高至97%。建设国家民族地区职业教育综合改革试验区，加快建设职教园区共享实训区，发展中高职院校共同体，推进产教深度融合，提升职业教育服务产业发展能力。

　　深化职业院校国际交流与合作，建设广西职业教育国际化试点和先行市，培养更多高素质“蓝领”人才。

　　促进文化大繁荣。培育和践行社会主义核心价值观，加强公民道德和精神文明建设，深入创建全国文明城市。传承和弘扬优秀传统文化，深入挖掘历史文化底蕴，推进历史文化名镇、名街、民族特色村落和历史建筑保护性开发利用，保护好文物和非物质文化遗产，推动三江侗族村寨申报世界文化遗产，支持融水、三江申报国家文化生态保护实验区。实施文化精品战略，繁荣文艺创作，打造艺术 品牌，培育发展柳州特色文化。加快构建现代公共文化服务体系，保障人民群众基本文化权益。完善基层公共文化设施，推动公共文化服务向基层倾斜、向农村延伸，丰富群众文化生活。加快文化产业发展，推进华侨城等重大项目建设，大力发展出版印刷、广告传媒等产业，提升文化综合实力。

　　统筹发展各项社会事业。创建“健康城市”，积极倡导全民健康生活方式，丰富提升国家卫生城市内涵。

　　加大医疗卫生投入，理顺城市社区卫生管理体制，提高新农合市级统筹试点、参保、筹资、补偿等保障水平。

　　不断提高妇女常见病筛查率，努力达到80%的目标，切实抓好艾滋病等重大传染病防控工作。促进全市人口长期均衡发展，重点解决好出生人口性别比偏高问题。积极推进体育惠民，进一步推动公共体育场馆对社会免费或低收费开放，深入开展市民徒步走、自行车绿色出行和龙舟竞赛等全民健身活动，办好2015年世界水上极速运动大赛、中国柳州国际内河帆船赛等重大体育赛事，积极引进社会资本，促进体育产业加快发展。

　　四、推进依法行政，建设法治政府

　　党的十八届四中全会明确提出，把“深入推进依法行政，加快建设法治政府”作为全面推进依法治国的一项重大任务。我们要深入贯彻依宪治国、依法治国的总方略，进一步增强法治思维、法制理念，针对执法程序不够规范，执法随意性较大和行政不作为、乱作为等问题，切实从以下几个方面推进依法行政，加快建设法治政府：

一是依法全面履行政府职能。坚持职权法定，落实行政组织和行政程序法律制度，推进机构、职能、权限、程序、责任法定化。各级政府和部门要坚持法定职责必须为、法无授权不可为，不得法外设定权力。要依据有关法规逐一界定权力依据、实施主体、职权范围、工作流程、监督方式等事项，着手制定权力清单。各级政务服务中心要按照行政审批事项和政务服务事项集中办理的要求，统一公布行政审批事项目录，更好为群众服务。

　　加快筹备政府立法，进一步清理政府规范性文件，顺利实现政府规范性文件与地方立法的有效衔接。抓好立法项目论证，科学确定立法项目和立法计划。广泛听取意见，探索政府立法征求意见机制和公众意见采纳情况反馈机制。切实增强公职人员依法履职意识，加快建立并公开责任清单，分级细化责任归属，落实具体责任主体，正确处理好依法依规行政和提高行政效能的关系，勇于担当、敢于负责、善于作为。

　　二是健全依法决策机制。各级政府及部门要严格执行《广西壮族自治区重大行政决策程序规定》，按照决策调研、咨询论证、公众参与、风险评估、合法性审查、集体讨论决定、公布结果的程序，作出重大行政决策。

　　重大行政决策事项提交集体讨论前，应当进行合法性审查。未经合法性审查或者审查不通过的，行政机关不能进行决策。政府重大行政决策由政府法制部门负责合法性审查，政府部门重大行政决策由部门法制机构负责合法性审查。重大行政决策事项涉及经济社会发展全局和群众切身利益的，应当进行社会稳定风险评估。建立健全政府法律顾问制度，发挥好法律顾问在推进依法行政中的积极作用。

　　三是深化行政执法体制改革。探索建立综合执法制度，在食品药品、工商质监、公共卫生、资源环境、农林水利等领域开展综合执法，着力解决重复执法、交叉执法等问题，完善部门协调配合机制，形成强大整体工作合力。建立行政执法评议考核机制，将评议结果作为考核领导干部和行政执法人员绩效的重要依据。健全城市管理综合执法机制，严格实行行政执法人员持证上岗和资格管理制度，认真执行罚缴分离和收支两条线管理制度。

　　四是坚持严格规范公正文明执法。

　　严格依照法定程序执法，明确行政执法具体操作流程。严格执行重大执法决定合法性审查制度、行政执法告知制度和执法全过程记录制度，健全行政执法调查取证程序和证据规则。严格规范自由裁量权的行使，防止执法随意性。全面落实行政执法责任制，对有违法或者不当执法行为的单位及个人，严格问责追究。建立公共法律服务体系，开展执法事前事后法律服务，评估执法效果。

　　五是强化对行政权力的制约和监督。进一步加强行政监察工作，自觉主动接受人大依法监督、政协民主监督，关注和重视社会各界和舆论监督，促进廉政建设。建立常态化监督机制，增强监督合力和实效。加强对政府内部权力的制约，推进财政资金分配使 用、国有资产监管、政府投资、政府采购、公共资源转让、公共工程建设等权力集中的部门和岗位的监督管理，实行分事行权、分岗设权、分级授权，定期轮岗，强化内部流程控制，防止权力滥用。保障和支持审计机关依法独立行使审计监督权，对公共资金、国有资产、国有资源和领导干部履行经济责任情况实行审计全覆盖。

　　六是全面推进政务公开。全面推进决策公开、执行公开、结果公开，做到依法、及时、准确、具体。各级政府及部门要依据权力清单，向社会全面公开政府职能、法律依据、管理流程等事项，重点推进财政预算、公共资源配置、社会公益事业建设等领域政府信息公开；对涉及公民、法人或其他组织权利和义务的规范性文件，依照法定程序予以公布。加强网上办事大厅和基层政务服务信息化平台建设，大力推进市、县（区）、乡镇政府网络互联互通。

　　各位代表！龙城正逢腾飞势，风劲恰是扬帆时。让我们紧密团结在以习近平同志为总书记的党中央周围，在市委的坚强领导下，与全市人民一道，改革创新，昂首前行，加快打造西江经济带核心城市，奋力谱写无愧于时代无愧于人民的豪迈诗篇！
